# 6DoF assembly pose estimation dataset for robotic manipulation

**DOI:** 10.1016/j.dib.2024.110834

**Published:** 2024-08-14

**Authors:** Kulunu Samarawickrama, Roel Pieters

**Affiliations:** Automation Technology and Mechanical Engineering, Tampere University, 33720 Tampere, Finland

**Keywords:** Assembly, Pose, Manipulation, Point clouds, Registration

## Abstract

Robotic assembling is a challenging task that requires cognition and dexterity. In recent years, perception tools have achieved tremendous success in endowing the cognitive capabilities to robots. Although these tools have succeeded in tasks such as detection, scene segmentation, pose estimation and grasp manipulation, the associated datasets and the dataset contents lack crucial information that requires adapting them for assembling pose estimation. Furthermore, existing datasets of object 3D meshes and point clouds are presented in non-canonical view frames and therefore lack information to train perception models that infer on a visual scene. The dataset presents 2 simulated object assembly scenes with RGB-D images, 3D mesh files and ground truth assembly poses as an extension for the State-of-the-Art BOP format. This enables smooth expansion of existing perception models in computer vision as well as development of novel algorithms for estimating assembly pose in robotic assembly manipulation tasks*.*

Specifications Table

**Table:** 

Subject	Computer Science.
Specific subject area	Cognitive Robotics.
Type of data	Simulated, RGB Image, 3D Mesh, Depth Image.
Data collection	Data were generated using the gazebo simulator [9] with 3D mesh files of assemblies obtained from thingyverse database [6,7]. The images were captured through a simulated RealSense D435i camera following hemisphere sampling procedure.
Data source location	Tampere University, Tampere, Finland.
Data accessibility	Repository name: 6DAPose - Synthetic Assembly Pose Dataset. Data identification number: 10.5281/zenodo.10117869 Direct URL to data: https://doi.org/10.5281/zenodo.10117869 Instructions for accessing these data: Download from graphical interface
Related research article	Samarawickrama, K., Sharma, G., Angleraud, A., & Pieters, R. 6D Assembly Pose Estimation by Point Cloud Registration for Robot Manipulation. IEEE International Conference on Automation Science and Engineering, 2024, in press*.*

## 1 Value of the Data

• Assembling is a demanding skill in robotic manipulation often addressed as a perception problem. We present a dataset of 2 assemblies simulated in a tabletop scene with information required for training and inference of perception based deep learning model that can endow assembling skills to a robot manipulator.
• The existing datasets [2,5] in research contain only geometric information and do not accurately represent information from a robotic perception environment. In contrast, our dataset presents multiple view samples of a tabletop assembly scene acquired through a depth sensor with relevant ground truth information.
• The fellow research community can easily adopt the data generation pipeline for any object assembly without limitation to a certain category of objects (e.g.: Furniture, mechanical components etc.). The dataset is formatted as an extension to the BOP format [3] which is the state-of-the-art benchmark for 6D pose estimation of objects. Furthermore, the dataset can further be utilized for benchmarking different assembly pose estimation techniques as the ground truth labels are provided.

## 2 Background

The purpose of this dataset is to produce features required to learn the spatial relationships and assembling sequence among objects in an assembly. These features must be extracted from the environment using sensor inputs in a real robotic application. In contrary to 3D mesh files and point cloud datasets which define an assembly in an arbitrary coordinate frame, a depth sensor can observe only a partial view of the object with respect to its coordinate frame. Therefore, an assembling scene viewed through a RGBD sensor fixed on a robot manipulator produce more accurate representation of a robotic assembly scene as presented in this dataset. The capabilities of modern physics simulators to simulate objects and camera sensors with definable parameters were utilized to produce the simulated dataset efficiently. We trained and benchmarked 6DAPose [1] which is an assembly pose estimation framework for robotic assembling based on this dataset.

## 3 Data Description

The dataset consists of the following object assemblies:

Table 1, Table 2

**Table 1:** Fidget gear Assembly [6].

Mesh name	Mesh Model	Diameter (cm)
Bottom casing		3.81
Left gear		2.78
Right gear		2.78
Bottom casing		3.82
Complete assembly

**Table 2:** Nema17 reducer assembly [7].

Mesh Name	Mesh Model	Diameter (cm)
Nema17 Motor		7.67
Sun Gear		2.70
Housing		6.17
Carrier		3.56
Cover		5.38
Complete Assembly		

**Fig. 1:**
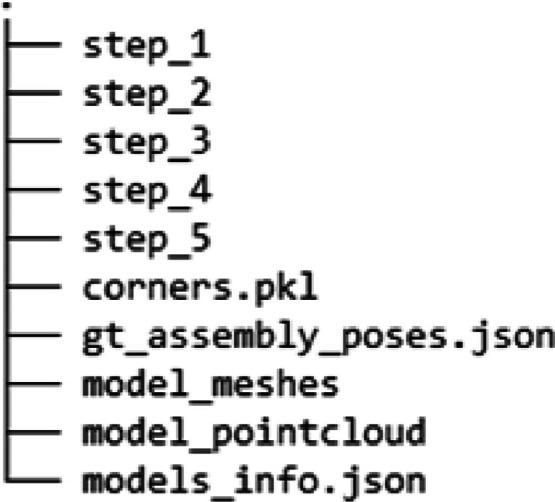
Dataset directory level 1 (root).

The directory architecture of the dataset is extended from BOP format. The directory level 1 (root) of the dataset is structured as described in Fig. 1.

• step_1, step_2, … directories contain information corresponding to each assembly step of the assembly.
• corners.pkl stores the 8 corners of a bounding box containing 3D mesh for all objects (row number corresponds to object id).
• gt_assembly_poses.json defines the assembly pose of each object with respect to base object. (This is an optional file only required in data generation process).
• model_meshes contains triangle mesh files of all objects in the assembly.
• model_pointcloud contains 3D point clouds of all objects.
• model_info.json is an optional file generated using BOP Toolkit [8] for pose error calculations.

In each assembly step directory, there N samples of simulated assembly scenes. Each assembly scene contains information in following directory structure.

• rgb directory contains 8-bit color images saved in .png format.
• depth directory contains 16-bit depth images saved in .png format.
• mask directory contains grayscale images of object silhouettes in .png format.
• seg_maps directory contains pixelwise segmentation labels of the scene as NumPy arrays.
• scene_camera.json file contains the following rgbd camera parameters as keys for each assembly scene.
  ◯ *dpt_cam_K*: depth intrinsic matrix of the depth camera (row-wise).
  ◯ *cam_K*: intrinsic matrix of the color camera (row-wise).
  ◯ *depth_scale*: multiplication factor to obtain depth in mm.
  ◯ *cam_R_w2c*: color optical frame rotation matrix with respect to world (row-wise).
  ◯ *cam_t_w2c*: color optical frame translation matrix with respect to world (row-wise).
  ◯ *dptopt_cam_R_w2c*: depth optical frame Rotation matrix with respect to world (row-wise).
  ◯ *dptopt_cam_t_w2c*: depth optical frame translation matrix with respect to world frame (row-wise).
• scene_gt.json contains ground truth 6DoF pose labels for each object in the scene.
  ◯ *obje_id*: object identifier.
  ◯ *cam_R_w2c*: object rotation matrix with respect to color optical frame (row-wise).
  ◯ *cam_t_w2c*: object translation matrix with respect to color optical frame (row-wise).
• scene_w_gt.json contains the same information as scene_gt.json with respect to world coordinate frame.
• Both dataset and dataset generation scripts are publicly available.
• Dataset is available to download at https://doi.org/10.5281/zenodo.10117869.
• Instructions for custom data generation is hosted in the repository at https://github.com/KulunuOS/6DAPose.

## 4 Experimental Design, Materials and Methods

The assembly scenes were simulated using gazebo classic physics simulator [9].

1. CAD file preprocess:
  The CAD files of all the objects in an assembly were acquired from opensource CAD archive thingyverse.com. CAD files were preprocessed to be compatible with the simulation by conversion to Polygon File Format (.ply). These files were further transformed to Simulation Description Format (.SDF) with texture properties. In an offline process involving human input, the assembly sequence and 6DoF assembly pose labels were annotated.
2. Assembly scene simulation:
  In order of assembly sequence, the objects and partially assembled objects were randomly placed on a table simulated on the origin of the simulation space. The table was 1 m high and the surface was white color. A single spotlight source was simulated above the assembly scene. The objects were placed on the most stable position under gravity (Fig. 2).

**Fig. 2:**
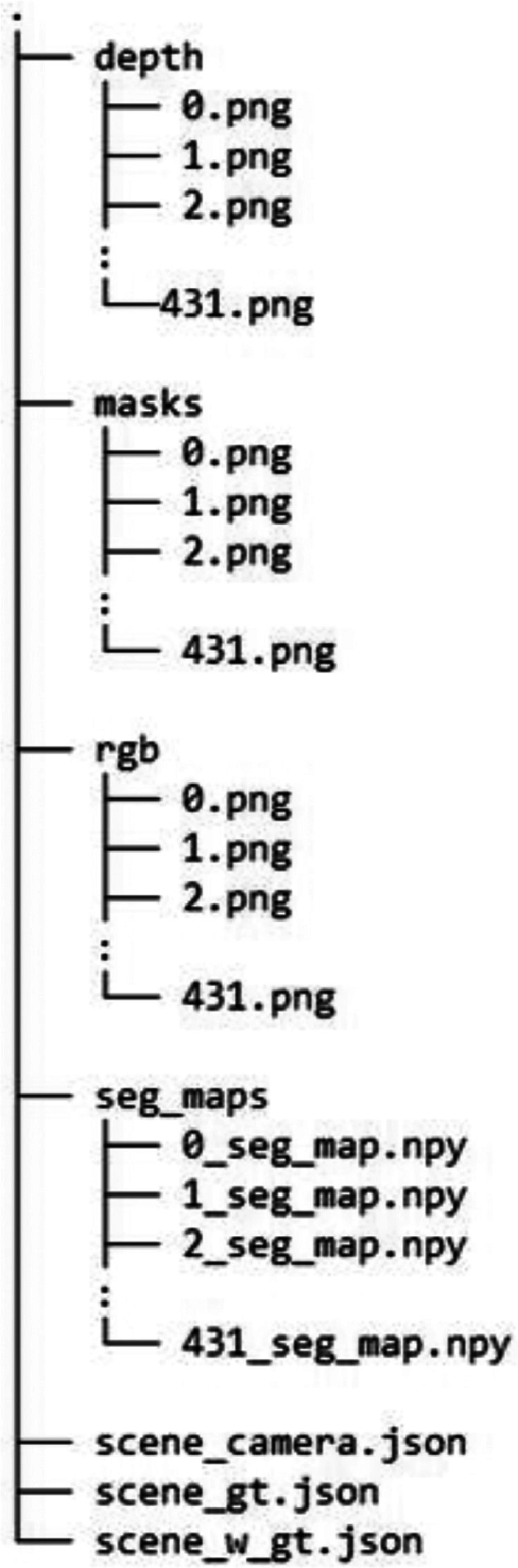
Dataset directory level 2.
3. RGBD sensor simulation:
  Gazebo simulator is associated with Robotic Operating System (ROS Noetic) framework and simulates sensors using plugins and Universal Robot Description Files (URDF). We use the opensource RealSense ROS plugin implemented by pal-robotics [10] and RealSense robot description [11]. RealSense D435i camera has multiple optical frames separately for color sensor, depth sensor and camera body and ROS tf_tools library publishes transformations between these frames which is important for recording data accurately,(Fig. 3).

**Fig. 3:**
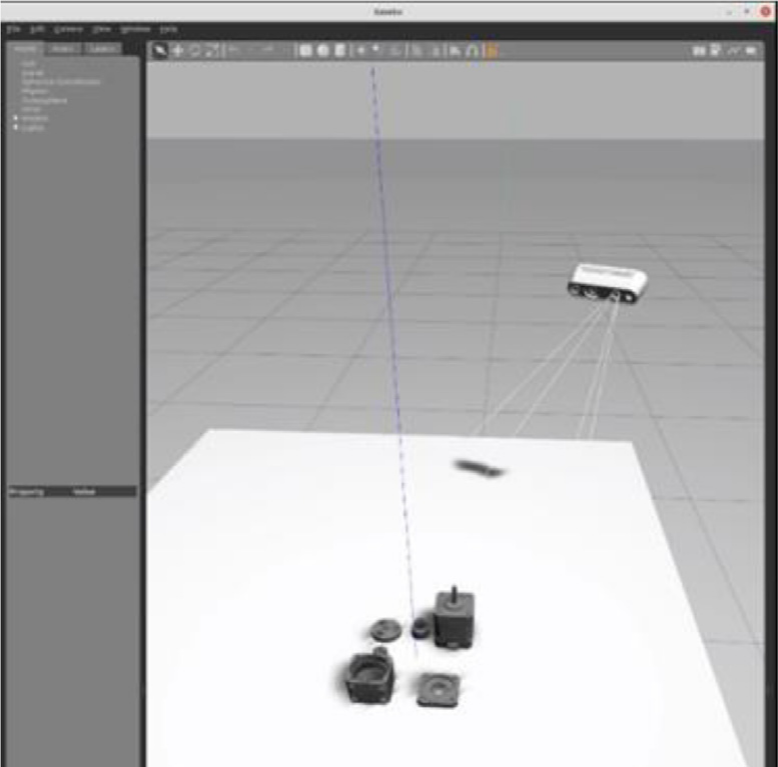
Simulation of RealSense D435i camera in gazebo classic.
4. Data capturing algorithm:
  We control the position of the simulated RGB-D sensor and sample viewpoints from an upper hemisphere centered around the origin of the simulation space following the hemisphere sampling algorithm of [4]. At each parametrized view sample, we record the information following the Algorithm 1 described below.

**Table:** **Algorithm 1** Assembly dataset generation.

**Parameters**: ϕ: Yaw angle of the camera. θ: Pitch angle of the camera. s: Scale of the camera. **Inputs**: 3D mesh models of the Assembly
**Procedure**: 1. Define and record assembly constraints. 2. *for* each assembly step: *for* each incremental value of ϕ, θ, s: Record i. I_RGB_ (color image) ii. I_D_ (depth image) iii. I_S_ (segmentation map) iv. P_obj_ (Ground truth 6DoF pose of objects) v. P_cam_ (Ground truth 6DoF pose of camera) vi. K_cam_ (Ground truth camera parameters
**Outputs**: I_RGB,_ I_D,_ I_S,_ P_obj,_ P_cam,_ K_cam_

Table 3 provides a summary of a single assembly (Nema17 reducer assembly) from dataset:

**Table 3:** Raw data sample from Nema17 assembly dataset.

## Limitations

Firstly, we annotate the assembly steps with human expertise rather than exhaustively checking for collisions in simulation. This is suitable when the assembling order and poses have only a single solution. In the presence of multiple correct assembly steps and pose configurations it is optimal to follow assembly-by-disassembly concept while checking for collisions to annotate the dataset [5].

Secondly, the sim-to-real gap in simulated data is considerable due to the limitations of the capabilities of gazebo classic simulator. This could be overcome by implementing the same procedure with ignition gazebo (gazebosim.org). However, realistic color images are only important when the pose estimation algorithms rely heavily on color image features.

## Ethics Statement

Authors declare that our work follow the ethical requirements for publication in Data in Brief and we confirm that our work does not involve human subjects, animal experiments, or any data collected from social media platforms.

## CRediT Author Statement

**Kulunu Samarawickrama:** Concept, Programming, Methodology, Results and Writing. **Roel Pieters:** Supervision, validation, review, and writing.

## Data Availability

6DoF Assembly Pose Estimation dataset for Robotic Manipulation (Original data) (Zenodo).
